# Mycotic popliteal aneurysm in an HIV-positive patient: a case report

**DOI:** 10.11604/pamj.2025.52.188.49454

**Published:** 2025-12-29

**Authors:** Grégoire Kouakou Ayegnon, Mohamed Diané, Samuel Kouamé Abro, Alassane Binaté, Ismaël Kouadio N'guessan, Christophe Gueu Meneas, Florent Kouakou Diby, Ambroise Loa Gnaba, Evelyne Pinnin Ouattara, Fatouma Sall, Anicet Kassi Adoubi

**Affiliations:** 1 Cardiovascular and Thoracic Surgery Unit, Bouaké University Hospital, Bouaké, Ivory Coast

**Keywords:** Mycotic aneurysm, HIV, surgery, CT angiography, case report

## Abstract

Mycotic popliteal aneurysm is rare. It is observed less rarely in patients immunocompromised by HIV. Its pathogenesis is linked to the weakening of the artery's angiotropism by HIV and to easy systemic mycobacterial colonisation. The diagnosis is based on clinical observations and computed tomography (CT) angiography of the limbs, which shows a mycotic pseudoaneurysm in the form of a cup-shaped eumycetoma and perianeurysmal abscesses. Surgery remains the treatment of choice, associated with intensive etiological treatment. We report the case of a 49-year-old HIV-positive patient admitted to Bouaké University Hospital (Ivory Coast) for a pre-ruptured claudicating popliteal vascular mass with critical ischaemia. A popliteal mycotic aneurysm was diagnosed by pelvic limb CT angiography, and the pathology report concluded that it was a eumycetoma.

## Introduction

Mycotic aneurysm is an abnormal, circumscribed dilation of the arterial wall resulting from various parietal alterations that may be local, caused by a systemic agent, or general, caused by a bacterial, fungal or viral agent [1] The angiotropism of HIV has been reported in various ways [2], with postulates of a high proportion of arterial aneurysms in HIV-immunocompromised individuals [3] We report a rare case of mycotic popliteal arterial aneurysm successfully operated on in an HIV-1-positive patient receiving antiretroviral therapy.

## Patient and observation

**Patient information:** the patient was a 49-year-old married male teacher who had been HIV-1 positive for eight years and was receiving irregular antiretroviral treatment (TDF+3TC+DTG).

**Figure 1:**
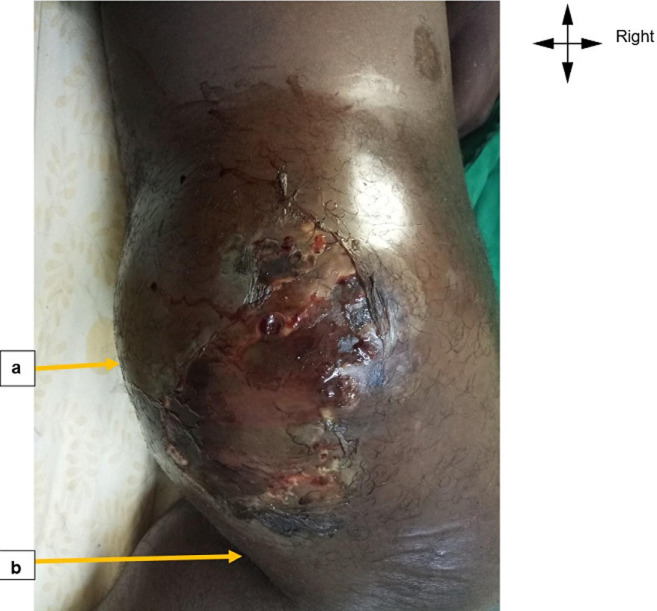
photograph of the right knee: A) a bulge with partial skin necrosis opposite the right popliteal region in an HIV-1-positive patient; B) right popliteal fossa

**Clinical findings:** the general examination showed that the patient was in fair general condition, with a slight fever, a body mass index of 27 kg/m^2^ and cyanosis of the right leg. Clinical examination of the right lower limb revealed a painful swelling on the posterior aspect of the lower third of the right thigh, which was pulsatile, non-expanding, warm, with a medium systolic murmur and hyperchromic dermohypodermitis extending from the toes to the right popliteal fossa. The skin covering the swelling was necrotic (Figure 1). The tumour measured 30cm in the long axis and 23cm in the short axis. The right subgonal portion below the vascular tumour was cold and non-oozing. There was no spontaneously visible entry point. Vascularly, the right pedal and retro-malleolar pulses were attenuated and dicrotic. Cardiac examination revealed an irregular heart rhythm with no additional sounds. Spleno-lymph node examination revealed right inguinal lymphadenopathy and painless Hackett II-type splenomegaly. Examination of the pleuropulmonary, digestive, neurological, osteoarticular, and urogenital systems was unremarkable.

**Chronology of the current episode:** he reported intermittent claudication with a gradually decreasing walking distance from 400 to 200 metres, associated with increasing, painful swelling of the lower third of the right thigh, above the right popliteal fossa, which had appeared three weeks before his admission to the cardiovascular surgery department of Bouaké University Hospital.

**Figure 2:**
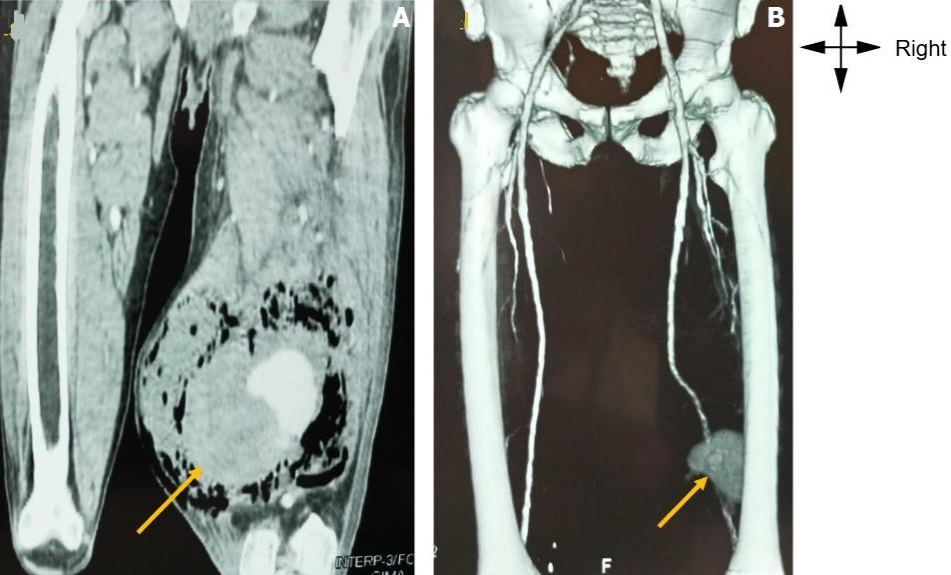
angiography of the pelvic limbs with IV injection of iodinated contrast medium: A) at the arterial phase in coronal and frontal; B) reconstructions showing a vascular mass, with a large haematoma and perianeurysmal abscesses, clearly limited to the appearance of a mycotic pseudoaneurysm developed at the expense of the right popliteal artery

**Diagnostic assessment:** the electrocardiogram showed sinus rhythm with polymorphic ventricular extrasystoles. Angiography of the lower limbs, including the coronal and reconstructive sections shown in Figure 2A and B, revealed an aneurysm of the popliteal artery with perianeurysmal muscle abscesses. The complete blood count showed anaemia with a haemoglobin level of 9.6 g/dl and leukoneutropenia at 2.9,000/ml. The viral load was 2.5 Log/ml. The CD4 count was 250/mm^3^. Pathological examination of the excised mass revealed a eumycetoma.

**Diagnosis:** right popliteal mycotic aneurysm in pre-rupture complicated by critical limb ischaemia.

**Figure 3:**
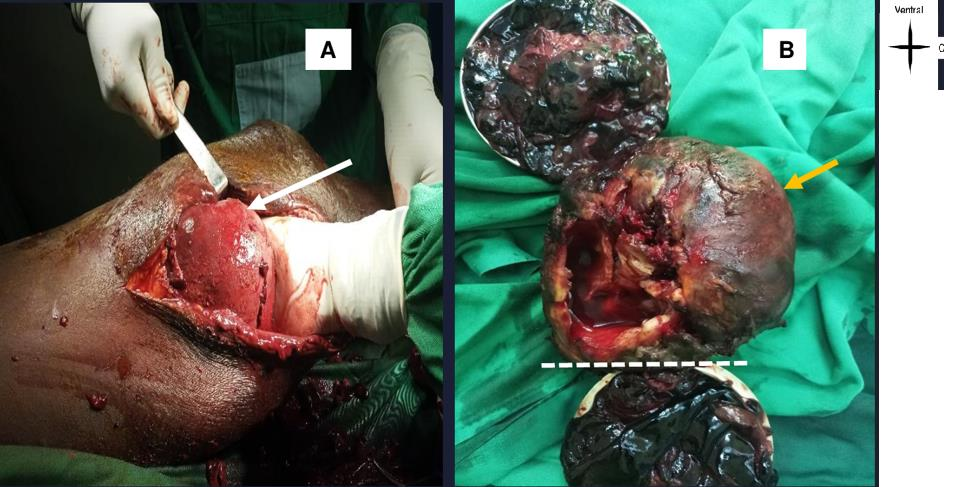
intraoperative view of a mycotic pseudoaneurysm in a patient seropositive for HIV-1; spongy mycotic mass (eumycetoma (c): A) 12cm in diameter, tangential to the popliteal artery; B) containing a 1,800g clot haematoma

**Therapeutic interventions:** the surgical procedure consisted of a popliteal aneurysmectomy involving the removal of a large cup-shaped spongy mass, shown in Figure 3A, which contained 1,800 g of clots of varying ages (Figure 3B). Arterial continuity was restored by interposing an inverted saphenous vein graft, secured by a double end-to-side arterial anastomosis, preceded by ligation of the proximal and distal ends of the right femorotibial artery.

**Follow-up and outcomes of the procedures:** the immediate postoperative period was uneventful. The patient's recovery was marked by functional recovery, allowing him to resume his social and professional activities.

**Patient's perspective:** “I hope to regain the functionality of my limb after surgery”.

**Informed consent:** the patient's informed consent was obtained.

## Discussion

Mycotic popliteal aneurysms are rare thanks to advances in antimicrobials and early management of systemic infections [1]. They are described in clinical cases. These aneurysms account for 3% of atheromatous and non-atheromatous aneurysms [2]. They pose a diagnostic and therapeutic challenge and occur in the context of systemic immunosuppressive disease, particularly HIV infection, which is associated with vascular complications [2-4]. These mycotic aneurysms have been recognised as unique clinical entities affecting HIV patients at a relatively young age, due to the high prevalence of HIV in this age group, which is sexually active and exposed to risk behaviours [2]. The young age of our patient corroborates the data in the literature. Although the pathogenesis of HIV-related aneurysms remains unclear, vasculitis has been observed in many HIV-infected patients, suggesting that chronic inflammation and endothelial dysfunction may be responsible for aneurysm formation [2].

Other theories include HIV infection of arterial smooth muscle cells, molecular mimicry, and bacterial infection. The latter involves four different mechanisms: contiguous septic processes extending to the periarterial lymphatic vessels and vasa vasorum of neighbouring arteries; bacterial infection of an intimal lesion or atherosclerotic plaque during bacteraemia; direct bacterial inoculation during arterial trauma; and septic embolisation reaching the vasa vasorum [2,4,5]. These postulates can be explained by our patient's long history of HIV infection, his irregularity and non-compliance with treatment, which are determining factors favouring aneurysms and thus supporting the theories mentioned above. Added to this is the increased susceptibility to opportunistic infections in immunocompromised individuals. However, even though the mechanisms involved in bacterial infection could not be established with certainty in our patient, we nevertheless suggest the hypothesis that bacterial infection could be strongly associated with chronic inflammation and pre-existing endothelial dysfunction related to HIV. These pathogenic mechanisms transform mycotic aneurysms into *"pseudoaneurysms"* of the popliteal artery. Discovered in the acute stage, they are characterised by painful, pulsatile and throbbing swelling of the right leg, accompanied by intermittent claudication in patients with an infectious disease. This vascular swelling is associated with a proven or occult infectious focus [1,4]. As in our patient, Dua *et al*. [6] found apyrexia in 70% of cases, with similar symptoms, associated with a portal of entry located in the lower limb. The clinical peculiarity of our patient lies in the discovery of a popliteal mycotic aneurysm at the stage of complications, such as critical distal limb ischaemia due to arterial thrombosis.

In addition, a complication of pre-rupture of the aneurysm, characterised by pain and shiny skin, was observed. At this stage, hyperkalaemia and systemic microthrombi related to the thrombosed mycotic aneurysm were observed. These ischaemic complications explain the polymorphic ventricular extrasystoles observed in our patient. However, the definitive diagnosis of popliteal mycotic aneurysm requires imaging techniques such as CT angiography, colour Doppler ultrasound and magnetic resonance imaging [4]. CT angiography of the limbs reveals a particular anatomopathological form of saccular aneurysm containing a haematoma limited by a shell and perianeurysmal abscess pockets, which is not described in the literature. This angiographic exception highlights the complexity of pathogenic variations in mycotic aneurysms in HIV-positive patients [2,7].

Furthermore, biological tests are of little help in definitively diagnosing mycotic popliteal aneurysms [6]. Indeed, the systematic antibiotic therapy prescribed for a swollen, painful and feverish leg reduces the chances of isolating the germs responsible for mycotic aneurysms, such as *Staphylococcus aureus, Salmonella* and occasionally *viridans* group streptococci, even though the infection is multi-bacterial in drug addicts and HIV-positive individuals [4,8]. In our case, no germs could be isolated, as reported in a series of cases [4]. Regarding immunosuppression, Orrapin *et al*. [9] reported that CD4 counts were not associated with the severity of vascular disease in HIV-infected patients. However, Silvestri *et al*. [10], as well as Nair *et al*. [7], reported that low CD4 counts were correlated with the occurrence of fungal aneurysms, regardless of their location [7,9,10].

Thus, the therapeutic challenge is not only surgical, but also medical. It is based on the treatment of sepsis, HIV retrovirus and ischaemia in order to prevent complications and reduce the risk of secondary amputation, which was avoided in our patient. Although various surgical techniques for popliteal aneurysms have been described, none are specific to mycotic aneurysms, let alone HIV-positive patients [3,6]. This is therefore a controversial subject that makes our therapeutic approach debatable, even though it improved both the functional prognosis of our patient's limb and his vital prognosis.

## Conclusion

In HIV-positive patients, popliteal mycotic aneurysms present as a febrile arterial mass prior to rupture. The presence of perianeurysmal haematomas and abscesses contributes to the diagnosis of a pseudoaneurysm. Surgical intervention by aneurysmectomy with extraction of eumycetoma and clots, followed by restoration of arterial continuity, ensures a good functional prognosis for the limb and the survival of the patient under intensive medical treatment.
